# First person – Malabika Chakrabarti

**DOI:** 10.1242/dmm.045179

**Published:** 2020-06-03

## Abstract

First Person is a series of interviews with the first authors of a selection of papers published in Disease Models & Mechanisms, helping early-career researchers promote themselves alongside their papers. Malabika Chakrabarti is first author on ‘Targeted repression of *Plasmodium* apicortin by host microRNA impairs malaria parasite growth and invasion’, published in DMM. Malabika is a PhD student in the lab of Dr Shailja Singh at Jawaharlal Nehru University, New Delhi, India, investigating the functional roles of human microRNA in the pathogenicity of the malaria parasite.


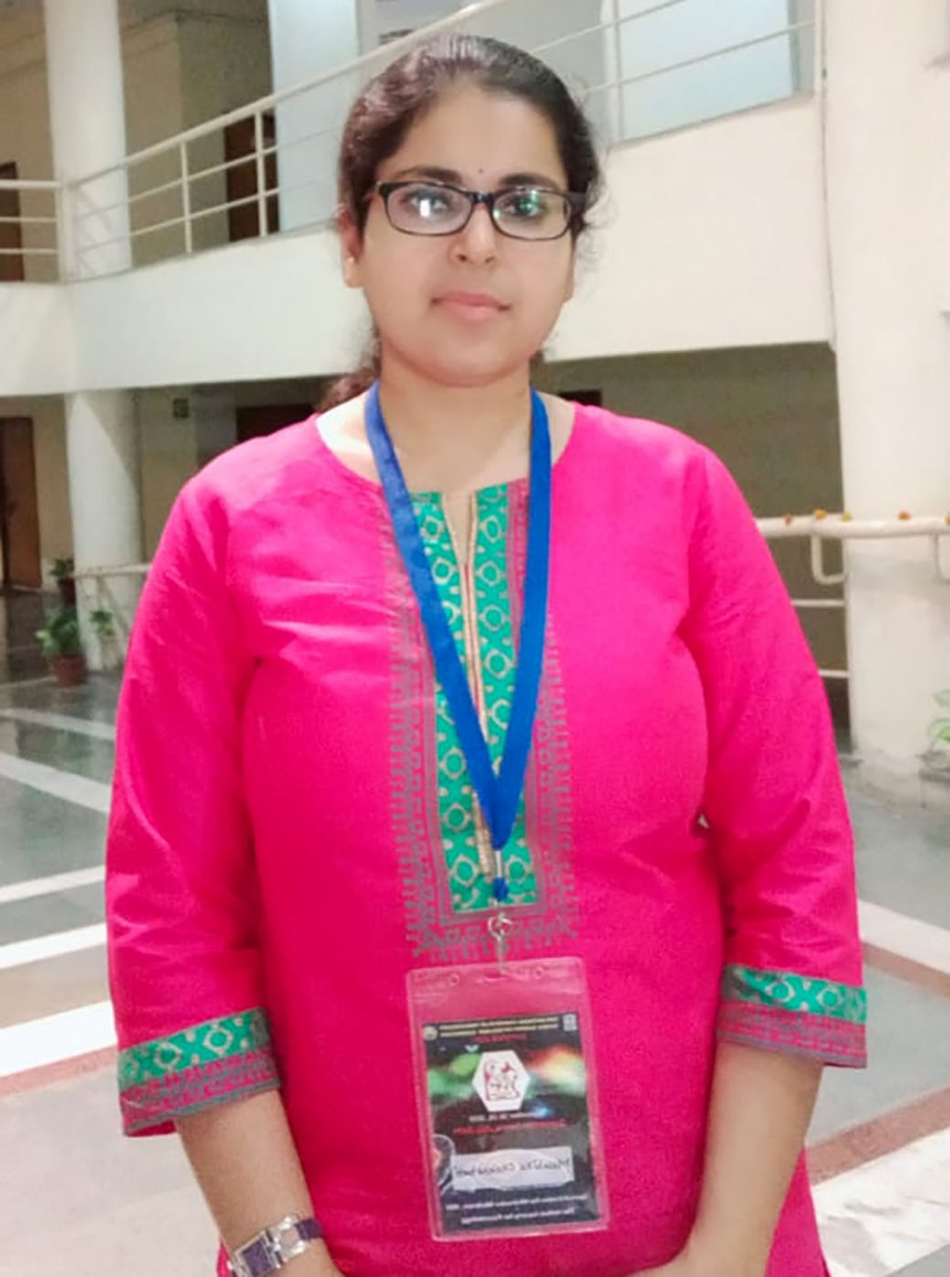


**Malabika Chakrabarti**

**How would you explain the main findings of your paper to non-scientific family and friends?**

Malaria is a serious disease causing massive lethal outcomes worldwide (especially in African countries and some Asian countries, including India). Research strategies to combat malaria include drugs and vaccines. Generic antimalarials have a serious problem of toxicity and hazardous side effects when applied on a regular basis. The development of non-toxic therapeutics is desperately needed in this scenario. Moreover, the emergence of parasite resistance to the generic antimalarial drugs is a significant concern. Our approach of developing host-inspired therapeutics involves the utilization of the human host's own physiological factors, such as microRNA (miRNA), for targeting the parasite. Therefore, the chances of toxicity or any adverse effect are reduced.

**What are the potential implications of these results for your field of research?**

Targeting the malaria parasite with novel and efficient therapeutics is always a challenge to the research community. The development of bio-therapeutics is an emerging area in this field. The development of peptide- or oligonucleotide-based therapeutics has always been a promising approach for drug discovery as the problem of emergence of resistance can be overcome, along with more specificity and less toxicity. MicroRNAs, being an important regulator of different biological processes of the eukaryotes (plants and animals), have been explored well for cancer research, for the development of biomarkers and gene therapy, but their role in infectious disease and host-pathogen interaction is still elusive. Also, the increasing level of malaria parasite resistance to the conventional antimalarials is demanding novel molecules with somewhat different mechanisms of action. Utilizing an erythrocytic miRNA pool for antimalarial activity is a novel approach of application of non-coding RNAs for the treatment of infectious disease, which would further help in understanding the role of miRNAs in host-pathogen interaction.

**What are the main advantages and drawbacks of the model system you have used as it relates to the disease you are investigating?**

We have used an erythrocytic disease model to study the condition *in vitro* by loading the human erythrocytes with miRNA mimics and subsequently infecting them with *Plasmodium falciparum*. Using this model, we could monitor the translocation of human miRNA into the parasites as well as its effect on parasite growth and invasion. The model actually replicates the natural condition of sickle-celled erythrocytes enriched with miRNAs and causing hindered growth and invasion of the parasite. Also, erythrocytes can be used as delivery systems of drug molecules and therapeutics *in vivo*. Considering these facts, the model is a useful tool in the development of bio-therapeutics. The drawback of this model is that it is only a model to study the effect of the bio-therapeutics *in vitro*. The *in vivo* study needs to be done separately.

“The efficiency of our miRNA-enriched erythrocyte model surprised me.”

**What has surprised you the most while conducting your research?**

The efficiency of our miRNA-enriched erythrocyte model surprised me. After loading of the cargo, we were a bit worried about the morphology and viability of the erythrocytes (whether they would be infected by the parasite). The stability of the miRNA mimics loaded inside the erythrocytes was another cause of concern. But the model worked out successfully and we could monitor the effect of miRNAs on the parasite along with other control cargos (DNA, miRNA inhibitor and scrambled miRNA mimic).

**Describe what you think is the most significant challenge impacting your research at this time and how will this be addressed over the next 10 years?**

In my opinion, the emergence of parasite resistance is the most significant challenge that might impact the overall drug development procedure against malaria. Chloroquine resistance has already spread in all the malaria-affected areas and the emergence of resistance to other antimalarials like sulfadoxine, pyrimethamine, mefloquine etc is also evident. Partial resistance to artemisinin has also been reported. In this situation, identifying diverse target moieties and developing novel therapeutics against them are the priorities in this field of research. In our work, we have tried to address the issue by utilizing the host factors against the parasite. In the future, developing host-inspired molecular medicine can be a novel approach to resolve the concern.

**What changes do you think could improve the professional lives of early-career scientists?**

Mentorship from senior scientists for improving scientific thinking is important and, in my case, my PI, Dr Shailja Singh, was instrumental in broadening the horizon for me to learn various techniques and access different facilities and instruments. Moreover, expertise in research comes through attending different conferences/seminars or workshops, where we can get acquainted with other researchers, exchange our views and ideas, and learn many things that are important for an early-career scientist.

**What's next for you?**

In this work, we have tried to monitor the human miRNA-mediated effect on the malaria parasite in an *in vitro* erythrocyte disease model. Executing the experiments in an *in vivo* model would complete the approach. Further, we have other miRNA-mRNA pairs to study and confirm their activity. In this way, we might be able to find other good targets in the parasite.
